# Results of emergency Hartmann's operation for obstructive or perforated left-sided colorectal cancer

**DOI:** 10.1186/1477-7819-6-90

**Published:** 2008-08-23

**Authors:** Pierre Charbonnet, Pascal Gervaz, Axel Andres, Pascal Bucher, Béatrice Konrad, Philippe Morel

**Affiliations:** 1 Department of Surgery, University Hospital Geneva, Switzerland

## Abstract

**Background:**

Up to 15% of colorectal cancer (CRC) patients present with obstructive or perforated tumours, and require emergency surgery. The Hartmann's procedure (HP) provides the opportunity to achieve a potentially curative (R0) resection, while minimizing surgical trauma in poor-risk patients. The aim of this study was to assess the surgical (operative mortality), and oncological (long-term survival after curative resection) results of emergency HP for obstructive or perforated left-sided CRC.

**Methods:**

A retrospective review of 50 patients who underwent emergency HP for perforated/obstructive CRC in our institution between 1995 and 2006.

**Results:**

Median age of patients was 75 (range 22–95) years and the indications for HP were obstruction (32) and perforation (18 patients). Operative mortality and morbidity were 8% and 26% respectively. 35 patients (70%) were operated with a curative intent; in this group, overall 1-, 3- and 5-year survival rates were 80%, 54% and 40%. In univariate analysis, the presence of lymph node metastases was associated with poor 5-year survival (62% [Stage II] vs. 27% [Stage III], log-rank test, p = 0.02). Eleven patients (22%) had their operation reversed with a median delay of 225 (range 94–390) days. In this subgroup, two patients died from distant metastases, but there were no instances of loco-regional recurrence.

**Conclusion:**

Hartmann's operation remains a good option to palliate symptoms in 30% of patients with left-sided CRC who are not candidates to a curative resection. For those who have a curative resection, the oncological outcome is acceptable, especially stage II patients, who appear to benefit the most from this surgical strategy.

## Background

Up to 15% of colorectal cancer (CRC) patients present with obstructive or perforated tumors and require emergency surgery. In this setting, colonic resections carry 10–20% mortality and 30–50 morbidity rates, due to the patients' poor condition [1,2]. Ideally, these patients would benefit from preoperative insertion of a metallic stent, in order to eventually perform a semi-elective curative resection with primary anastomosis [3]. Unfortunately, most of these procedures are performed out of hours, in elderly individuals, who are often dehydrated and hemodynamically unstable, due to concomitant sepsis [4]: under these conditions, many experienced surgeons would consider prohibitive the risk to perform a primary anastomosis. It is therefore not surprising that the operation described by Henri Hartmann in 1921, consisting of resection of the offending part of the left/sigmoid colon, proximal end colostomy and closure of the rectal stump, remains popular today, and has continued to extricate surgeons and patients alike from many a delicate situation [5].

This procedure gained wide acceptance in the 1970s for the management of complicated diverticulitis, and it is surprising that few series have focused on CRC patients, and addressed the oncological outcome of this procedure. Back in the early 80s, surgeons from the Mayo Clinic reported 54%, 23%, and 3% 5-year survival rates for Stage II, III and IV cancers respectively, but a majority of patients were electively operated [6]. Subsequently, Kristiansen reported 5-year survival rate of 31% and that intestinal continuity was restored in seven (24%) of 29 patients who underwent HP for obstructive left-sided CRC [7]. In addition, McArdle and Hole have demonstrated that emergency surgery for CRC is associated with high (8%) mortality and poor (39%) 5-year overall survival rates, even after a curative resection [8]. It would therefore be tempting to consider that emergency HP for left-sided CRC is an obsolete operation, often performed with a palliative intent in elderly and/or very sick patients with a high risk of cancer-related as well as intercurrent death [9].

Many surgeons, however, still consider that HP remains a good option to achieve R0 resection, while minimizing surgical trauma in poor-risk CRC patients [10,11]. The aim of this study was to assess the surgical (operative mortality), oncological (long-term survival after curative resection) and functional (permanent colostomy vs. restoration of intestinal continuity) results of emergency HP for obstructive or perforated left-sided CRC.

## Methods

This is a retrospective analysis of all patients who underwent emergency Hartmann's procedure for CRC in our institution between 1995 and 2006. The University Hospital of Geneva is the only public medical institution in a mainly urban area, and thus provides primary care for 75–80% of a population of 500,000 inhabitants. An average number of 350 colectomies are performed each year in our institution, 90–95 being emergency resections. Initially, we considered all patients who were operated within 48 hours of their unplanned admission for colonic occlusion or colorectal perforation. Subsequently, we selected in this population patients with a final diagnosis of colorectal adenocarcinoma, as determined by histopathologic examination of the surgical specimen. The charts of 50 consecutive patients with obstructive/perforated left-sided CRC who underwent emergency HP were analyzed.

The following parameters were included in the structured database:

**1) Patients' demographics**; gender; age; and ASA score,

**2) Tumour characteristics**; Location (left colon vs. rectum); mode of presentation (obstructive vs. perforated); TNM stage; and mode of dissemination for metastatic cancers (peritoneal vs. liver).

**3) Modalities of HP (first stage)**; type of resection (curative vs. palliative); degree of peritoneal contamination (none vs. purulent vs. stercoral); operative mortality, defined as death within 30 days of surgery; and postoperative complications. The operative report was assessed to determine with precision the reasons for not having performed a primary anastomosis; those included preoperative co-morbidities, peroperative hemodynamic instability, localized/generalized peritonitis, and doubtful viability of the proximal colon.

**4) Modalities of HP reversal (second stage**); delay between HP and restoration of intestinal continuity; operative mortality; and surgical complications. We also recorded the preoperative imaging and endoscopic investigations performed prior to reversal, such as CT scan, colonoscopy, PET scan

Follow-up was carried out through routine visits at our Outpatient Surgical Oncology Clinic, for those patients who underwent adjuvant radiation or chemotherapy. Serum CarcinoEmbryonary Antigen (CEA) levels were assessed every three months during the first two years after surgery and every six months thereafter. Yearly colonoscopy and chest X-rays were performed routinely and abdominal CT scan or liver ultrasonography were performed in patients with raising CEA levels or clinical suspicion for tumour recurrence. Whenever possible, confirmation of data was obtained through interviews with the physicians or the patients. Primary outcome measure was overall survival; secondary outcome measures were: 1) disease-free survival; 2) surgical mortality; and 3) restoration of intestinal continuity (Hartmann's reversal).

### Statistical analysis

Life-tables curves (global survival endpoints: death, irrespective of course, and tumor-free survival endpoints: definite tumor recurrence or death) were analyzed with the Kaplan-Meier method and distributions were compared by the log-rank test. In case of simultaneous analysis of more than 2 populations, statistical differences were assessed by an extension of Gehan's generalized Wilcoxon test, Peto and Peto's generalized Wilcoxon test and the log-rank test algorithms, using the Statistica 5.5. software (Statsoft Inc, Tulsa, OK, US). Continuous data were analyzed by bilateral Student t test and dichotomous data were analyzed by chi-square test. P values lower than 0.05 were considered significant.

## Results

**Figure 1:**
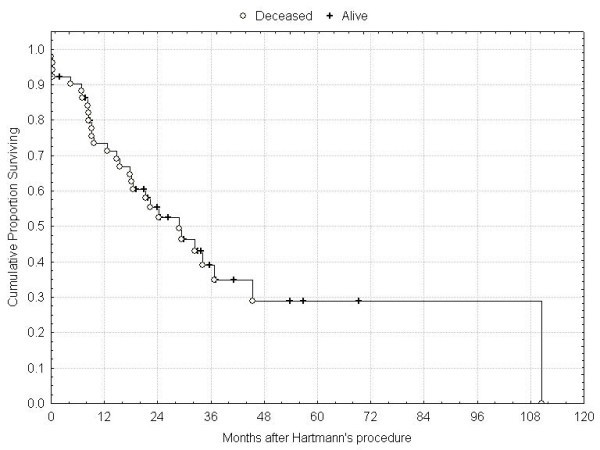
Overall survival.

**Figure 2:**
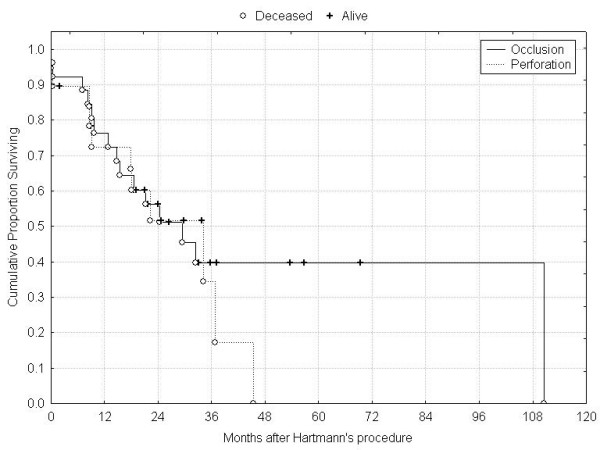
Survival according to type of resection.

**Figure 3:**
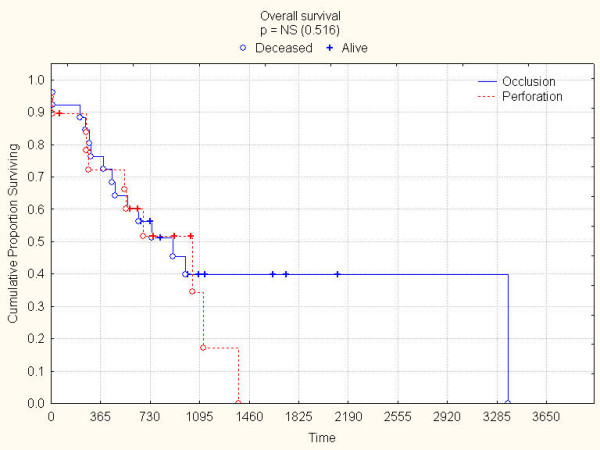
Overall survival according to mode of presentation.

**Figure 4:**
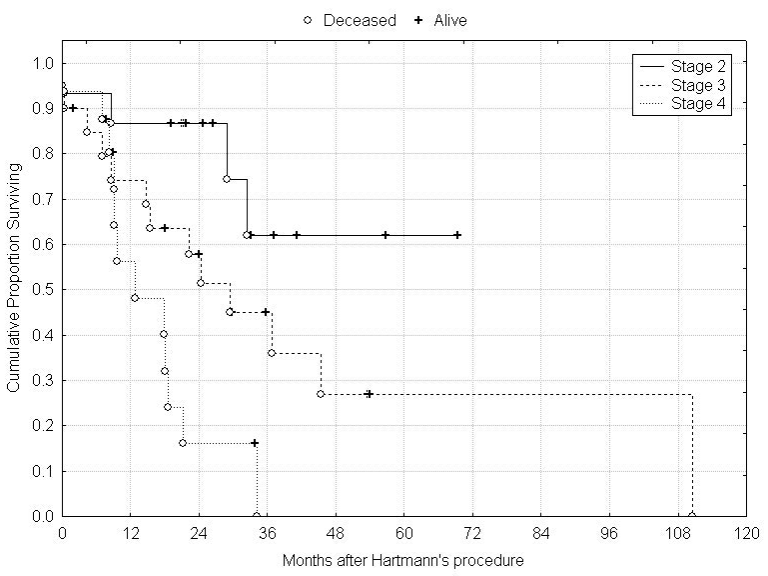
Overall survival according to tumour stage.

Median age of patients was 75 (range 22–95) years and the indications for HP were obstruction (32) and perforation (18 patients). The median follow-up was 22 (range 5–111) months. All fifty patients were available for complete follow up, except one who left our country. 29 patients died during this study period, and at the time of last follow-up, 5 patients were alive with recurrence. Operative mortality and morbidity were 8% and 26% respectively. Patients' and tumours characteristics are summarized in Table 1. Fifteen patients presented with metastatic disease (12 = liver and 3 = carcinomatosis). For the whole group, overall 1-, 3-, and 5-year survival rates were 72%, 38% and 30% (Figure 1). 35 patients (70%) were operated with a curative intent, with a median survival of 28 months; in this group, overall 1-, 3- and 5-year survival rates were 80%, 54% and 40% (Figure 2). In univariate analysis, the mode of presentation (perforation vs. obstruction) was not associated with improved survival (p = 0.51) (Figure 3). By, contrast, the presence of lymph node metastases was associated with decreased 5-year survival (62% [Stage II] vs. 27% [Stage III], log-rank test, p = 0.02) (Figure 4).

**Table 1:** Patients' and Tumour Characteristics (N = 50)

**Parameter**	
**Gender**	
Male	24
Female	26
**Age, median (range)**	75 (22–95)
**Tumour location**	
Colon	35
Rectum	15
**Tumour stage**	
II	13
III	21
IV	16
**Adjuvant treatment**	
None	31
Radiation therapy	3
Chemotherapy	16
**Restoration of intestinal continuity**	
No	39
Yes	11
**Cause of death (N = 29)**	
Cancer	23 (local recurrence = 6)
Postoperative	4
Non cancer-related	2

Eleven patients (22% for the whole group, but 31% of patients operated with a curative intent) had their operation reversed with a median delay of 225 (range 94–390) days. There were no death and no anastomotic dehiscence after the second stage of the procedure. However, two patients had unsuccessful attempt to restore intestinal continuity, one because of dense adhesions within the pelvis, the other because of local recurrence, which was undetected prior to surgery. In this subgroup, two patients eventually died from distant metastases.

## Discussion

The data presented here indicate that 70% of patients who underwent emergency surgery for obstructive/perforated left-sided CRC had a curative resection. In this group, 5-year survival rate was 40%. The prognosis was similar to elective procedures, and strongly related to tumour stage, more than to the mode of presentation. For stage II patients, 5-year overall survival rate was 62%, and Hartmann's reversal rate was 63%. For those patients who presented with Stage IV disease, HP was effective in palliating symptoms during a median survival of only 13 months.

In accordance with population-based study from Burgundy [12], our data demonstrate that, in this difficult clinical setting, resection for cure is still possible in 70% of cases. By contrast, the operative mortality (8%) and morbidity rates (26%) in this series compare favourably with other, reporting mortality rates in the 10–15% range for similar patients and conditions [13-15]. It has been recognized, however, that the negative impact of emergency surgery on CRC outcome is confined to the immediate postoperative period [16]. Among Stage II-III CRC patients surviving surgery, there is little difference in overall survival between patients undergoing emergency compared with elective operation [17]. Thus, the goals of surgery in poor-risk patients with obstructive or perforated CRC are two-fold; 1) providing effective palliation of symptoms in patients with R1–R2 resections: and 2) minimizing surgical mortality in patients with R0 resections.

As rightfully pointed out by Armbruster [18], primary resection with anastomosis and HP are not competing operations, but two situation-dependent therapeutic alternatives. It should, however, be noted that the performance of a resection with primary anastomosis exposes the patients to the risk of anastomotic dehiscence; and that a leaking colorectal anastomosis is associated with a significant increase in local recurrence [19,20], as well as poor long-term survival [21,22]. Therefore, efforts should be made to avoid this complication and its consequences, such as wound infection, intra-abdominal sepsis and the need for subsequent re-operation, which inevitably delay administration of postoperative chemotherapy in Stage III patients, who would benefit the most from this adjuvant modality [23].

It is known that a high percentage of CRC patients who underwent HP end up with a permanent stoma. In our series, eleven patients only (22% for the whole group; 31% of patients operated with a curative intent) had their operation reversed with a median delay of 225 (range 94–390) days. In two additional patients reversal was attempted, but was considered unfeasible at the time of surgery. Similarly low reversal rates have been reported by other groups [24,25]. In our institution, Hartmann's reversal in patients with CRC is usually delayed for 8–10 months, but not more: experience from the Dutch Rectal cancer Trial has shown that if a stoma was not closed within the first year, it would probably become permanent [26]. The interval between the two stages of the procedures allows for identification of good risk patients for stoma closure; patients with stage II tumours; patients with stage III cancers who subsequently underwent adjuvant chemotherapy; and socially active patients. By contrast, elderly patients with T4 or N2 tumours, who are at high risk for developing local recurrence, are candidates for a definitive colostomy.

## Conclusion

Hartmann's operation is effective in palliating symptoms in 30% of patients with obstructive/perforated stage IV left-sided CRC. For those who are candidates to a curative resection, this approach minimizes surgical mortality/morbidity and is associated with stage-dependent survival rates close to those of elective operations. Patients with stage II cancers have good oncological (62% 5-year survival rate) and functional (63% reversal rate) outcomes, and benefit the most from this surgical strategy. Some experts consider that the Hartmann's procedure is today "out of vogue"; it might be true for complicated diverticulitis, but probably not for the emergency management of left-sided colorectal cancer-the original indication for this time-honoured operation.

## Competing interests

The authors declare that they have no competing interests.

## Authors' contributions

PC and PG conceived of the study and wrote the manuscript. AA performed the statistical analysis. FG and BK coordinated the study and helped to draft the manuscript. PM supervised the study. All authors read and approved the final manuscript.
